# Statistical considerations for repeatability and reproducibility of quantitative imaging biomarkers

**DOI:** 10.1259/bjro.20210083

**Published:** 2022-08-22

**Authors:** Shangyuan Ye, Jeong Youn Lim, Wei Huang

**Affiliations:** 1 Biostatistics Shared Resource, Knight Cancer Institute, Oregon Health and Science University, Portland, OR, United States; 2 Advanced Imaging Research Center, Oregon Health and Science University, Portland, OR, United States

## Abstract

Quantitative imaging biomarkers (QIBs) are increasingly used in clinical studies. Because many QIBs are derived through multiple steps in image data acquisition and data analysis, QIB measurements can produce large variabilities, posing a significant challenge in translating QIBs into clinical trials, and ultimately, clinical practice. Both repeatability and reproducibility constitute the reliability of a QIB measurement. In this article, we review the statistical aspects of repeatability and reproducibility of QIB measurements by introducing methods and metrics for assessments of QIB repeatability and reproducibility and illustrating the impact of QIB measurement error on sample size and statistical power calculations, as well as predictive performance with a QIB as a predictive biomarker.

## Introduction

Medical imaging modalities such as CT, MRI, and positron emission tomography (PET) are routinely used in clinical practice for disease screening, diagnosis, staging, therapeutic monitoring, evaluation of residual disease, and assessment of disease recurrence. Traditionally, image contrast-based qualitative interpretations of medical images are the most commonly employed radiology practice. With advances in imaging technologies in recent years, imaging metrics that can quantify tissue biological and physiological properties, in addition to those that quantify tissue morphology such as disease size, are increasingly used in research and early phase clinical trials to characterize disease and response to treatment. A recent review^1^ by a group of principal investigators from the Quantitative Imaging Network (National Cancer Institute, National Institutes of Health) has called for wider incorporation of quantitative imaging methods into clinical trials, and eventually, clinical practice for evaluation of cancer therapy response. In the emerging era of precision medicine, quantitative imaging biomarkers (QIBs) can be integrated with quantitative biomarkers from genomics, transcriptomics, proteomics, and metabolomics to facilitate patient stratification for individualized treatment strategy and improve treatment outcome.^2^ A QIB is defined as “an objective characteristic derived from an in vivo image measured on a ratio or interval scale as an indicator of normal biological processes, pathogenic processes or a response to a therapeutic intervention.”^3^ QIBs can be generally classified into five different types: structural, morphological, textural, functional, and physical property QIBs.^4^ Kessler et al^3^ have introduced terminologies related to QIBs for scientific studies. Study designs and statistical methods used for assessing QIB technical performances have been extensively reviewed.^4–8^ Because many QIBs are derived through multiple steps in image data acquisition and data analysis that often involve different manufacturer scanner platforms and different computer algorithms and software tools, QIB measurements can produce large variabilities, which pose a significant challenge in translating QIBs into clinical trials, and ultimately, clinical practice. In order for a QIB and its changes to be interpretable in clinical settings across institutions and clinics for disease characterization and therapy response assessment, it is highly important to evaluate the repeatability and reproducibility of the QIB.

Both repeatability and reproducibility constitute the reliability of a QIB measurement. Repeatability refers to the precision of a QIB measured under identical conditions (*e.g.* using the same measurement procedure, same measurement system, same image analysis algorithm, and same location over a short period of time; also known as repeatability condition),^3,4^ which is mainly a measure of the within-subject variability and the variability caused by the same imaging device over time. On the other hand, reproducibility refers to the precision of a QIB measured under different experimental conditions^3,4^ (also known as the reproducibility condition), which is mainly a measure of the variability associated with different measurement systems, imaging methods, study sites, and population. In recent years, many studies have been conducted to investigate the reliability of different QIBs. For example, Yokoo et al^9^ studied the precision of hepatic proton-density fat-fraction measurements by using MRI; Lodge^10^ examined the repeatability of standardized uptake value (SUV) in oncologic^11^F-fludeoxy glucose (FDG) PET studies; Shukla-Dave et al^12^ reviewed and emphasized the need for assessment of reproducibility and repeatability of MRI QIBs in oncologic studies; Park et al^13^ reviewed the challenges in reproducibility of quantitative radiomics metrics; Fedorov et al^14^ and Schwier et al^15^ assessed the repeatability of radiomics features from multiparametric MRI of small prostate tumors; Hernando et al^16^ quantified the reproducibility of MRI-based proton-density fat-fraction measurements in a phantom across vendor platforms at different field strengths; Kalpathy et al^17^ investigated the repeatability and reproducibility of tumor volume estimate from CT images of lung cancer; Lin et al^18^ evaluated the repeatability of ^18^F-NaF PET–derived SUV metrics; Baumgartner et al^11^ studied the repeatability of ^18^F-FDG PET brain imaging; Jafer et al.,^19^ Winfield et al.,^20^ Weller et al.,^21^ Lecler et al,^22^ and Lu et al^23^ estimated the repeatability and reproducibility of the apparent diffusion coefficient (ADC) derived from diffusion-weighted MRI; Hagiwara et al^24^ studied the repeatability and reproducibility of quantitative relaxometry with a multidynamic multiecho MRI sequence using a phantom and normal healthy human subjects; Jafari-Khouzani et al^25^ appraised the repeatability of brain tumor perfusion measurement using dynamic susceptibility contrast MRI; Han et al^26^ studied the repeatability and reproducibility of the ultrasonic attenuation coefficient and backscatter coefficient in the liver; Hagiwara et al^27^ reviewed the repeatability of MRI and CT QIBs; Wang et al^28^ examined the repeatability and reproducibility of 2D and 3D hepatic MR elastography markers in healthy volunteers; and Olin et al^29^ investigated the reproducibility of the MR-based attenuation correction factors in PET/MRI and its impact on ^18^F-FDG PET quantification in patients with non-small cell lung cancer.

In this article, we review the statistical aspects on repeatability and reproducibility of QIB. We introduce methods and metrics for assessment of QIB repeatability and reproducibility, and illustrate the impact of QIB measurement error on sample size and statistical power calculations, as well as performance as a predictive biomarker.

## Measurement error model

Precision of a QIB is defined as the closeness of agreement between repeated measurements of the QIB,^3^ and repeatability and reproducibility comprise different sources of variability that may impact the precision of a given QIB. Measurement error is defined as the difference between a measured quantity and its true value.^30^ Any sources of variability can cause measurement errors in QIB measurements. Though it is critical to identify and obtain valid inference on the impact of every component of variation (see Section 3), we start by introducing a general model on measurement error. Table 1 lists commonly used symbols in this article.

**Table 1.** List of symbols and notations

~	Distributed as
N(a,b)	Normal distribution with mean a and variance b
df	Degree of freedom
$\chi_{df}^{2}$	Quantile function of chi-square distribution
Var	Variance
$Y_{itl}$	Measured QIB value from the l th measurements of a repeated QIB measurement made at time t for subject i
$X_{i}$	True QIB value for subject i
n	Number of subjects included in the study
m	Number of replicates for each subject
μ	Mean of X
ϵ	Measurement error
δ	Within-subject error (under repeatability condition)
γ	Between-condition error (under reproducibility condition)
γδ	Interaction between subject and condition
$\sigma_{X}^{2},{\sigma_{\epsilon }^{2},\sigma }_{\delta }^{2}$ , $\sigma_{\gamma }^{2}$ , $\sigma_{\gamma \delta }^{2}$	Variance of X , ϵ , δ , γ , and γδ , respectively
${\hat{\sigma }}_{\epsilon }^{2}$	ˆ Represents the estimator of a parameter
$H_{0}$	Null hypothesis
$H_{1}$	Alternative hypothesis

QIBs, quantitative imaging biomarkers.

In a measurement error model, instead of the true QIB value, we can only observe QIB values with errors that are random across different QIB measurements. If the errors are constant for all measurements, then the error is called bias.^3^ Since both repeatability and reproducibility mainly concern random errors, we assume that there is no bias and that the random errors are independent and identically distributed with the mean equal to zero and variance of $\sigma_{\epsilon }^{2}$ . Thus, the only unknown parameter $\sigma_{\epsilon }^{2}$ measures the level of variability, with larger values indicating larger variability or worse precision. Let *Y_itl_* be the measured QIB value from the *l*th measurements of a repeated QIB measurement made at time *t* for subject *i* with *X_it_* being the corresponding true value, the measurement error model can be expressed as

(1) $$Y_{itl}=\beta_{0t}+\beta_{1}X_{it}+\epsilon_{itl},\epsilon_{itl}∼N(0,\sigma_{\epsilon }^{2}),$$

where *β*_0*t*_ represents the bias of the QIB measurement and *β*_1_ represents the proportional bias of the QIB measurement. The random error $\epsilon_{itl}$ is assumed to be normally distributed (other commonly used distribution assumptions are discussed below). Because both bias and proportional bias cannot be identified solely through the QIB measurements, it is usually assumed that these values are constant and known in advance from ground-truth studies such as a phantom study.^31^ Because we can always standardize the QIB measurement through 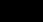 to remove the effect of bias and proportional bias, without the loss of generalizability, we can reasonably assume the setting of no bias and no proportional bias (*β*_0*t*_ = 0 and *β*_1_ = 1), and the model (1) becomes

(2) $$Y_{itl}=X_{it}+\epsilon_{itl}.$$

## Repeatability and reproducibility

Repeatability and reproducibility represent different sources of variability (causing measurement error $\epsilon_{itl}$ ) in model (1). Repeatability refers to the precision of a QIB measured under identical conditions, while reproducibility refers to the precision of a QIB measured under different experimental conditions.^3,4^ Kessler et al^3^ recommends identifying and evaluating each experimental component separately that contributes to reproducibility-related variability. However, in many real-life settings, it is very difficult to independently investigate each source of variability under the reproducibility condition. Thus, the statistical model introduced below only assumes a single parameter representing all sources of variability associated with the reproducibility condition.

Following the measurement error model (2), we consider a simplified setting where all measurements for subject *i* are made at a relatively short time interval so that the true value *X_i_* remains unchanged. That is, let *Y_ijk_* be the *k*th repeated QIB measurement made on subject *i* under experimental condition *j* (different experimental conditions may include different measurement systems, imaging methods, and countries/regions), in a similar fashion to model (1) we employ a linear relationship between *Y_ijk_* and *X_i_*,^4,5,32^ but further breaking down the measurement error $\epsilon_{ijk}$ into different components of repeatability- and reproducibility-related errors. Similar to model (2), we assume there are no bias or proportional bias, and the model that accounts for both repeatability- and reproducibility-related errors can be written as

(3) $$Y_{ijk}=X_{i}+\delta_{ik}+\gamma_{j}+(\gamma \delta {)}_{ij},\delta_{ik}∼N(0,\sigma_{\delta }^{2}),\gamma_{j}∼N(0,\sigma_{\gamma }^{2}),\;(\gamma \delta {)}_{i}j∼N(0,\sigma_{\gamma \delta }^{2})$$

The terms *δ_ik_*, *γ_j_*, and (*γδ*)*_ij_* represent different components of measurement error caused by within-subject variability (under repeatability condition), between-condition variability (under reproducibility condition), and the interaction between subject and condition, respectively, and we assume they follow normal distributions. In general, the random error variances $\sigma_{\delta }^{2}$ , $\sigma_{\gamma }^{2}$ , and $\sigma_{\gamma \delta }^{2}$ are the key performance characteristics used in repeatability and reproducibility studies (see details below).

### Repeatability

Many studies have been conducted to investigate the repeatability of QIBs for different imaging modalities, including but not limited to CT,^17,18^ MRI,^9,12,14,15,20–25^ and PET.^10,11,18,29^ Test–retest studies are usually performed to evaluate the repeatability of QIB measurements. These studies usually require each subject to be scanned repeatedly over a short period of time with the assumption that *X_i_* does not change. If the repeatability study condition is held, *i.e*. all repeated scans are performed at the same location, with the same measurement procedure, and using the same measurement system and image analysis algorithm, an estimate of QIB repeatability can be calculated.

In practice, test–retest studies can be performed fairly easily using a phantom, but could be difficult with human subjects due to expenses and logistic problems. Therefore, repeatability studies using human subjects are often limited to a small number of replicates (usually two) on each subject. Furthermore, for imaging studies with contrast administration, because there usually exists a required contrast washout period between two consecutive scans (*e.g.* consecutive dynamic contrast-enhanced (DCE) MRI scans are usually required to be performed at least 24 h apart),^4,31^ “coffee-break” experiments where there is only a short break between repeated scans are not always possible. Thus, for repeatability studies with long interval between scans, possible changes in the true values should also be considered in the model.

Specifically, for a test–retest study with *n* subjects and *m* replicates for each subject, since all experiments are conducted under the same experimental condition, without the loss of generalizability, we set *j* = 1 and model (3) becomes

(4) $$Y_{i1k}=X_{i}+\delta_{ik}+\gamma_{1}$$

for i=1,⋯,n and k=1,⋯,m . The random effect variance $\sigma_{\delta }^{2}$ is the key performance characteristic used in a repeatability study, with smaller value corresponding to better repeatability. Because we only consider QIB measurements in a single-site and single measurement system study for a test–retest repeatability study, the random error *γ_j_* in (3), which measures the reproducibility-related variability such as between-site variability, becomes a condition-specific systematic error *γ*_1_ in (4), representing the condition-specific bias. As discussed in Section 2, the constant bias *γ*_1_ cannot be identified through the test–retest study and should be estimated through a phantom study with ground-truth values. The term $(\gamma \delta {)}_{ij}$ in model (3) vanishes because there is only one study condition and the interaction effect cannot be observed.

Many metrics related to the estimate of within-subject variance $\sigma_{\delta }^{2}$ have been proposed to quantify the magnitude of repeatability.^4^ Table 2 shows the metrics considered in this article for repeatability measurement. The within-subject standard deviation (wSD) is the most commonly used metrics for assessing repeatability. It is the standard deviation ( $\sigma_{\delta }$ ) of repeat measurements for a single subject. If we assume all subjects have the same $\sigma_{\delta }$ and the ground true values *X_i_* are independent and normally distributed, wSD can be obtained by fitting a linear mixed-effects model with subject-specified random intercepts using maximum likelihood.^33^ Although other estimation procedures such as method of moment estimators can also provide consistent estimation of wSD, the maximum likelihood method is usually preferred for small sample size when model (3) is correctly constructed. Alternatively, wSD can be calculated by averaging the within-subject sample variances^32^

**Table 2.** Repeatability and reproducibility metrics

*Repeatability metrics*	
wSD	Within-subject standard deviation
ICC	Intraclass correlation coefficient, proportion of total variation associated with the variation of true value
wCV	Within-subject coefficient of variation, ratio of the within-subject standard deviation to its mean
*Reproducibility metrics*	
tSD (reproducibility SD)	Total standard deviation under reproducibility conditions
CCC	Concordance correlation coefficient, measurement agreement between two experimental conditions

(5) $$\hat{wSD}=\sqrt{\frac{1}{2}\sum_{i=1}^{n}\frac{1}{m-1}\sum_{k=1}^{m}(Y_{j1k}-{\bar{Y}}_{i}{)}^{2}}$$

where ${\overset{-}{Y}}_{i}=\frac{\sum_{k=1}^{m}‍Y_{i1k}}{m}$ . This estimator (equation (5)) is equivalent to the estimator obtained from the one-way analysis of variance (ANOVA) model.^34^

Another closely related metric for repeatability measurement is the intraclass correlation coefficient (ICC),^35,36^ which is defined as the proportion of total variation that is associated with the variation of true value. That is, if we assume $X_{i}∼N(\mu ,\sigma_{X}^{2})$ , we have $Y_{i1k}∼N(\mu +\gamma_{1},\sigma_{X}^{2}+\sigma_{\delta }^{2})$ and

(6) $$ICC=\frac{Var(X_{i})}{Var(Y_{i1k})}=\frac{\sigma_{X}^{2}}{\sigma_{X}^{2}+\sigma_{\delta }^{2}}.$$

Comparing the variance of QIB measurement $Y_{i1k}$ (denominator of equation (6)) with the variance of the true value *X_i_* (numerator of equation (6)), we can observe that the extra variation equals to the within-subject variation $\sigma_{\delta }^{2}$ . If $\sigma_{\delta }^{2}$ is much smaller than $\sigma_{X}^{2}$ , then $Var(Y_{i1k})\approx Var(X_{i})$ and ICC is close to 1, which indicates that the measurement error contributes little to variation of the QIB measurement. In other words, larger ICC implies better repeatability and smaller ICC implies worse repeatability. ICC can be estimated with known estimates of $\sigma_{\delta }$ and $\sigma_{X}$ (equation (6)), both of which can be obtained by either fitting a linear mixed-effects model with subject-specified random intercepts or one-way ANOVA model. When using ICC as the measure of repeatability, it is crucial to ensure that the subjects participating in the study are representative of the study population so that the estimated QIB variation can well reflect the variation of the study population (variance of $X_{i}$ ).

The within-subject coefficient of variation (wCV) (Table 2) is an alternative metric of wSD. The wCV is defined as the ratio of the within-subject standard deviation to its mean, which is commonly used for test–retest studies of repeatability when wSD is not constant among studied subjects and model (4) becomes inadequate. A useful alternative model for model (4) is to assume that the wSD increased proportionally with the true value *X_i_*, *i.e.,*

(7) $$Y_{i1k}=\delta_{ik}X_{i}+\gamma_{1},\delta_{ik}>0.$$

In model (7), with the extra constraint of $\delta_{ik}>0$ , it is more adequate to assume $\delta_{ik}$ follows log-normal or Weibull distributions^37^ with the mean of $\delta_{ik}$ equal to one. Raunig et al^4^ suggest using log-normal distribution so that the log-transformed QIB measurement $log(Y_{i1k})$ is normally distributed (after adjusting for the site-specific bias *γ*_1_)

(8) $$log\left(Y_{i1k}-\gamma_{1}\right)=log\left(X_{i}\right)+log\left(\delta_{ik}\right),log\left(\delta_{ik}\right)∼N\left(0,\sigma_{\delta^{`}}^{2}\right).$$

Under model (8), wCV only depends on the log-transformed within-subject variance $\sigma_{\delta `}^{2}$ ^4^ through the form

(9) $$wCV\;=\sqrt{e^{\sigma_{\delta^{\prime }}^{2}}-1}$$

Therefore, we can apply any of the estimators of wSD on the log-transformed QIB measurements to obtain valid estimates of $\sigma_{\delta `}^{2}$ , and wCV can be estimated by plugging the ${\hat{\sigma }}_{\delta `}^{2}$ into model (9). Without the log-normal distribution assumption, by mimicking estimator (5), when *m* = 2, we can still estimate wCV by pooling and averaging the within-subject sample coefficient variation^32^

(10) $$\hat{mCV}=\sqrt{\frac{1}{n}\sum_{i=1}^{n}‍\frac{1}{m-1}\sum_{k=1}^{m}‍\frac{{\left(Y_{i1k}-{\overset{-}{Y}}_{i}\right)}^{2}}{{\left({\overset{-}{Y}}_{i}\right)}^{2}}}.$$

Both ICC and wCV have the benefit of being dimensionless, which make them useful for comparing quantities measured on different scales.

Instead of a single point estimates of the repeatability metric (either wSD, ICC, or wCV), it is desirable to make inference on these values through either constructing confidence intervals (CIs) or performing hypothesis testing. Both confidence interval and hypothesis testing involve estimating the distribution of the estimator and thus depend on the choice of the estimation method. For wSD (denoted as *σ* and the corresponding estimator denoted as $\hat{\sigma }$ ), if the ANOVA-type estimators such as equations (5) and (10) are used, $n(m-1){\hat{\sigma }}^{2}/\sigma^{2}$ follows χ^2^ distribution with a degree of freedom (*df*) of n(m−1) . Thus, the (1 − *α*) ∗ 100% CI of $\hat{\sigma }$ is

(11) $$\left(\frac{\hat{\sigma }}{\sqrt{n(m-1)X_{df,1-\alpha /2}^{2}}},\frac{\hat{\sigma }}{\sqrt{n(m-1)X_{df,\alpha /2}^{2}}}\right)$$

where $\chi_{df,\alpha /2}^{2}$ and $\chi_{df,1-\alpha /2}^{2}$ are the α/2 and 1-α/2 quantiles, respectively, of the χ^2^ distribution with degree of freedom *df*. To test whether the level of wSD is greater than a threshold value *c*, we conduct hypothesis test with null hypothesis $H_{0}:\sigma^{2}\leq c^{2}$ *vs* alternative hypothesis $H_{1}:\sigma^{2}>c^{2}$ . The corresponding test statistic is $T=\frac{n(m-1){\hat{\sigma }}^{2}}{c^{2}}$ and we reject the null hypothesis if $T>\chi_{df,\alpha }^{2}$ . On the other hand, if the maximum likelihood estimators are used, making inference based on the asymptotic distribution of $\hat{\sigma }$ is usually problematic for small sample sizes and numerical methods such as bootstrap CI^38^ or profile likelihood CI^39^ should be considered. For wCV, CI of $\sigma_{\delta^{\prime }}^{2}$ on the log-transformed data is first determined using formula (11). Then the CI of wCV can be obtained based on model (9). Because the estimator of ICC is a nonlinear function of ${\hat{\sigma }}_{\delta }$ and ${\hat{\sigma }}_{X}$ , its exact sampling distribution is not available. In this case, bootstrap confidence intervals have been extensively used in literature.^40,41^ We can also construct the confidence interval of $\hat{ICC}$ by approximating its sampling distribution using either *F* (also known as Satterthwaite approximation) or β distribution.^42^ Based on Monte Carlo simulation of the sampling distribution of the generalized pivotal quantity of ICC, Ionan et al^43^ suggests the generalized confidence interval proposed by Weerahandi.^44^ Sample size calculation can be conducted using the method provided by A’Hern.^36^

### Reproducibility

Reproducibility concerns the consistency or precision of the QIB measurement made on the same subject with the same experimental design but under different experimental conditions, such as different measurement device. Reproducibility of QIBs for different imaging modalities, such as CT,^17^ MRI,^9,12,16,19,24^ and PET,^13,29^ have also been extensively studied. Although many experimental factors can be included for the reproducibility study condition, it is practically impossible to consider all conditions for a single reproducibility study. Raunig et al^4^ provided a list of conditions that can be tested in reproducibility studies. Depending on which condition is being tested, reproducibility studies can be classified into two categories: (1) repeated measurement design and (2) cohort measurement design. For example, the former can be used to study the variability caused by different scanners, while the latter can be used to study the variability caused by different study sites. Because the within-subject variability is generally embedded in the variability under different experimental conditions, a reproducibility study can generate repeatability results for each experimental condition. Specifically, for a repeated measurement design, subject *i* is repeatedly measured *m* times for each of the *J* experimental conditions; for a cohort measurement design, each subject will be repeatedly measured *m* times under one of the experimental conditions. That is, a repeated measurement design requires each subject being measured *m* × *J* times, while a cohort measurement design requires each subject only being measured *m* times. Model (3) is valid for both experimental designs, and the key performance characteristics is the sum of the random effect variances ( $\sigma_{\epsilon }^{2}=\sigma_{\delta }^{2}+\sigma_{\gamma }^{2}+\sigma_{\gamma \delta }^{2}$ ), which represents the total variation under the reproducibility study condition. However, subjects in the cohort measurement design are only measured under a single experimental condition and thus, the subject-condition interaction effect $(\gamma \delta {)}_{ij}$ does not exist. In this case, we can assume $\sigma_{\gamma \delta }^{2}=0$ , and the total variation for the cohort measurement design becomes $\sigma_{\delta }^{2}+\sigma_{\gamma }^{2}$ .

Similar to repeatability studies, either linear mixed effects models or two-way ANOVA can be used to fit the data and obtain valid estimates of $\sigma_{\delta }^{2}$ , $\sigma_{\gamma }^{2}$ , and $\sigma_{\gamma \delta }^{2}$ . The square root of the total variance $\sigma_{\epsilon }^{2}$ , denoted as total SD (tSD) (Table 2) or reproducibility SD, can be used as a metric to quantify the magnitude of reproducibility. If estimators based on linear mixed effects models are considered, the sampling distributions for these estimators are unknown and numerical methods such as bootstrap or permutation should be used to make valid inferences on these parameters. On the other hand, if ANOVA-type estimators are considered, all the three terms $df{\hat{\sigma }}_{\delta }^{2}/\sigma_{\delta }^{2}$ , $df{\hat{\sigma }}_{\gamma }^{2}/\sigma_{\gamma }^{2}$ , and $df{\hat{\sigma }}_{\gamma \delta }^{2}/\sigma_{\gamma \delta }^{2}$ follow χ^2^ distributions and the corresponding CIs and test statistics can be easily obtained. However, because the sampling distribution of ${\hat{\sigma }}_{\epsilon }^{2}$ , which equals to ${\hat{\sigma }}_{\delta }^{2}+{\hat{\sigma }}_{\gamma }^{2}+{\hat{\sigma }}_{\gamma \delta }^{2}$ , is unknown, numerical methods are recommended.

In a scenario where only two experimental conditions are being compared, *e.g.* comparing two scanner platforms, the variance of $\gamma_{j}$ ( $\sigma_{\gamma }^{2}$ ) is no longer estimable (only $\gamma_{1}$ and $\gamma_{2}$ exist). We then use the agreement between these two experimental conditions as the measure of reproducibility. For such a situation where *m* = 1 for a repeated measurement design, Lawrence and Lin^45^ proposed the concordance correlation coefficient (CCC) (Table 2) as an agreement measure of reproducibility,^46^ defined as

$$CCC=\frac{\sigma_{1}\sigma_{2}\rho_{12}}{\sigma_{1}^{2}+\sigma_{2}^{2}+(\gamma_{2}-\gamma_{1}{)}^{2}}$$

to evaluate the QIB agreement between the two experimental conditions, where $\sigma_{1}$ and $\sigma_{2}$ are the standard deviations of the measured QIB under experiment condition 1 and 2, respectively, and $\rho_{12}$ is the Pearson correlation between the measured QIB values $Y_{i1}$ and $Y_{i2}$ . Similar to Pearson correlation, CCC is also ranged between −1 and 1, with values close to 1 (or −1) representing good concordance (or good discordance) and 0 representing no correlation.

The method introduced by Lawrence and Lin^45^ is commonly used to estimate CCC and its CI.^47^ Lin and Williamson^48^ provide a simple method to perform sample size calculation of CCC.

### Plots for repeatability and reproducibility studies

**Figure 1.**
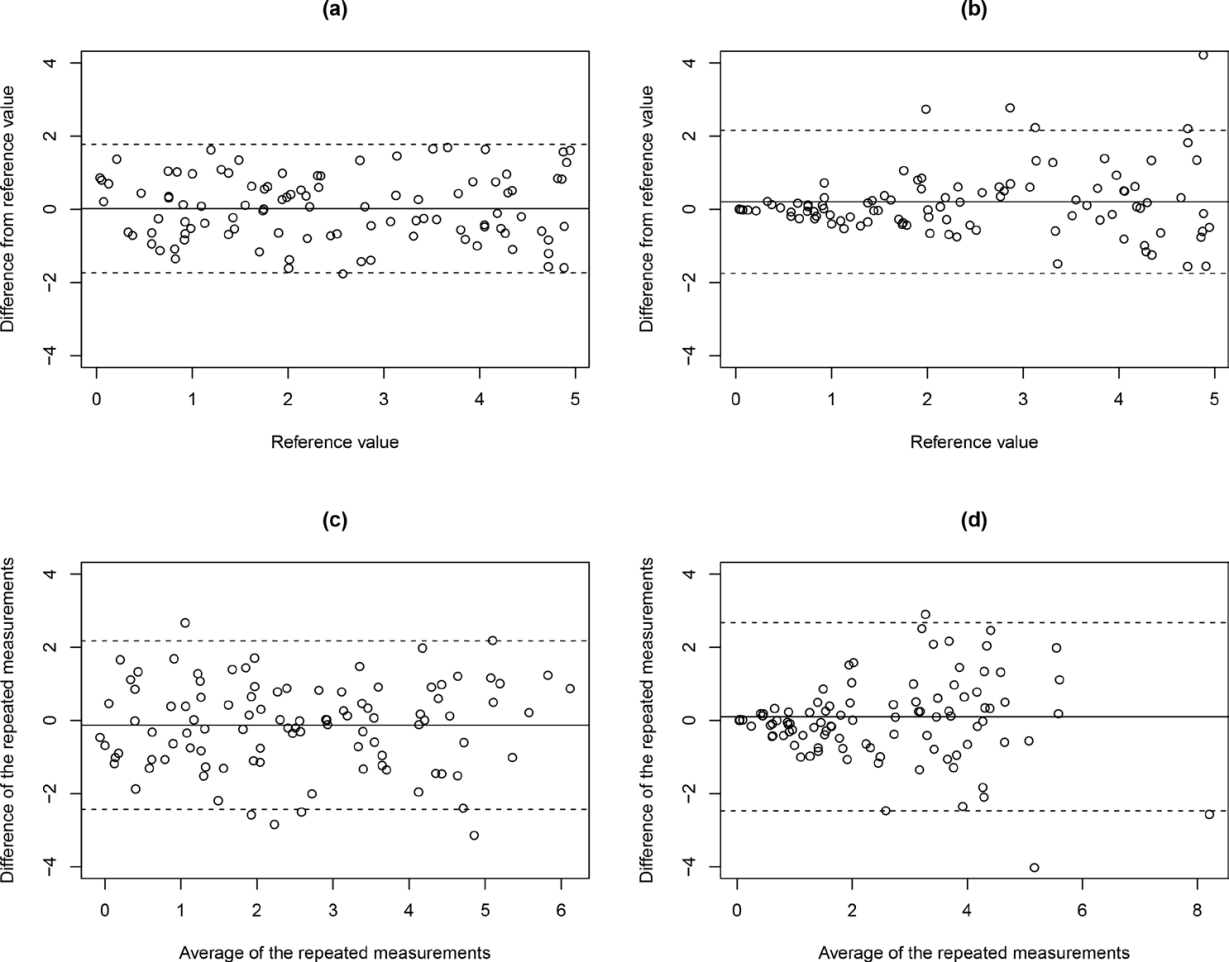
Bland–Altman plot examples for (**a**) reference value available with constant measurement error variance, (**b**) reference value available with increasing measurement error variance, (**c**) reference value not available with constant measurement error variance, and (**b**) reference value not available with increasing measurement error variance.

Varies plots can be used to visually study the impact of measurement errors on repeatability and reproducibility studies. For repeatability studies, although Box-Whisker plots can provide information on the variability for each subject,^4^ they are usually not feasible for test–retest studies since only few repeated measurements (usually two) are available for each subject. Bland–Altman plots^49^ are commonly included in repeatability studies to visualize trends in variability within measurement intervals. Figure 1 demonstrates the Bland–Altman plots based on 100 simulated data with *X* generated uniformly at random from interval 0 and 5. When gold-standard or reference values are available (see the setting described in Obuchowski et al^32^), the difference between the QIB measurements and the corresponding reference values are plotted against the reference values in Bland–Altman plots. Figure 1a illustrates the case of additive error (model (4) with *σ* = 0.8), while Figure 1b shows the case of multiplicative error (model (7) with *σ* = 0.3). When reference values are not available, *e.g.* test–retest studies, standard deviations (or differences for the case of *m* = 2) of repeated measurements are plotted against the averages of repeated measurements in Bland–Altman plots. Figure 1c and d show the cases of additive and multiplicative errors based on two repeated measurements and same values of *σ* as in Figure 1a and Figure 1b, respectively. When multiplicative errors are observed (*e.g.* in Figure 1(b) , and (d)), log-transformed QIB measurements should be considered as suggested in Section 3.1.

**Figure 2.**
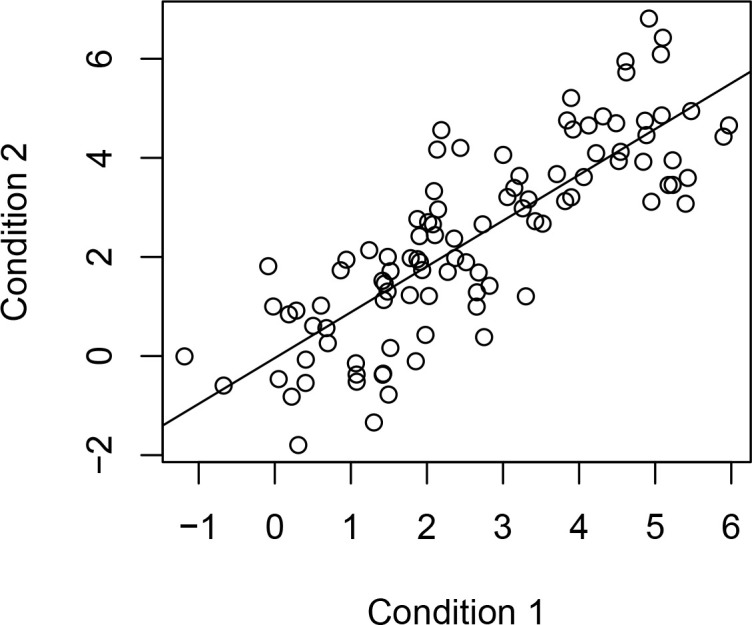
Scatter plot of two method comparison (slope = 0.831, intercept = 0.335).

When only two experimental conditions are being compared, Bland–Altman plots can also be used in reproducibility studies, especially when gold-standard or reference values are not available.^4^ In addition, scatter plot of experimental Condition 1 *vs* Condition 2 with a fitted regression line can provide useful visualization of the agreement between these two methods (Figure 2). When there exists more than two experimental conditions, we can follow the same procedure as above for each pair of the experimental conditions.

## Examples of the impact of QIB measurement errors on clinical studies

### QIB as trial end point

**Figure 3.**
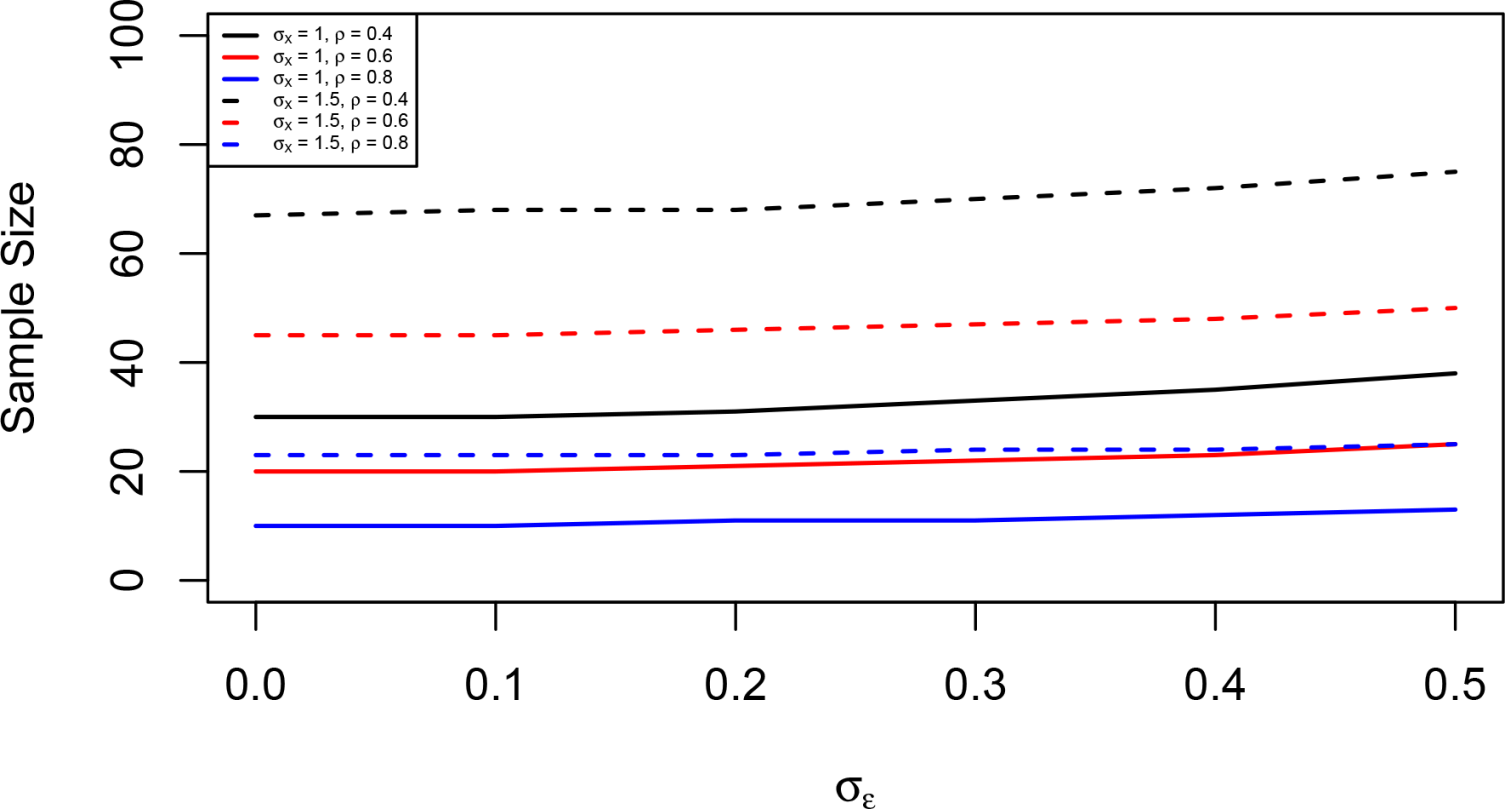
Sample size required to achieve 80% power with a significance level of 5% against measurement error standard deviation $\sigma_{\epsilon }$ . The true difference $\mu_{2}-\mu_{1}$ is 0.5, commonly represents 50% change for log-transformed QIB; ρ=0.4,0.6, or 0.8; $\sigma_{X}=1$ or 1.5; and c=0 . QIB, quantitative imaging biomarker.

QIBs can serve as a clinical trial’s endpoint to assess treatment efficacy, where subjects enrolled in the study are scanned before and after treatment, and the difference of the mean QIB measurements over the treatment course is used to determine the efficacy of the treatment. Since it is often nearly impossible to perform repeated measurements at a single time point in a longitudinal study, it is difficult to assess repeatability and/or reproducibility-related QIB measurement errors. From model (2), for a setting without repeated measurements, let $Y_{i1}$ and $Y_{i2}$ be the QIB measurements (or log-transformed QIB measurements) before and after intervention for subject i , respectively, and we further assume that the corresponding true value $X_{it}$ follows normal distribution with mean $\mu_{t}$ and variance $\sigma_{X}^{2}$ for t=1,2 and i=1,⋯,n . The common approach to assess treatment efficacy is to test if the mean difference is greater than a threshold value c so that the difference is practically meaningful (*i.e.* null hypothesis $H_{0}:\mu_{2}-\mu_{1}\leq c$ *vs* alternative hypothesis $H_{1}:\mu_{2}-\mu_{1}>c$ ). Under model (2), the corresponding test statistic *Z* is

$$Z=\frac{n-{}^{1}/2\sum_{i=1}(Y_{i2}-Y_{i1}-c)}{\sqrt{2}(1-\rho )(\sigma_{X}^{2}+\sigma_{\epsilon }^{2})}∼N(0,1)$$

where *ρ* is the Pearson correlation (ranged between 0 and 1) between $Y_{i1}$ and $Y_{i2}$ . Thus, for a statistically significant level of *α*, the formula of minimum sample size required to achieve *β* power is

(12) $$n=\left(\frac{\left(2(1-\rho )(\sigma_{X}^{2}+\sigma_{\epsilon }^{2})(z_{\alpha }+z_{\beta }\right)}{c-(\mu_{2}-\mu_{1})}\right)$$

where $z_{\alpha }$ and $z_{\beta }$ are the α and β quantiles of standard normal distribution. For example, under the setting of c=0 and $\mu_{2}-\mu_{1}=0.5$ , which represents 50% change against no change in hypothesis testing if log-transformed QIB is considered, Figure 3 illustrates the required sample sizes to achieve 80% power with a significance level of 5% for different values of $\sigma_{\epsilon }$ , $\sigma_{X}$ , and ρ . From both Figure 3 and equation (12), we can notice that the required sample size is an increasing function of $\sigma_{X}^{2}$ and $\sigma_{\epsilon }^{2}$ , and is a decreasing function of ρ .

For a longitudinal study, the correlation parameter ρ measures the level of dependence between the QIB measurements before and after treatment, with longer time interval between the measures generally resulting in smaller ρ . Obuchowski et al^6^ showed that the range of ρ , which depends on the time interval of the two measurements, is between 0 and $\frac{\sigma_{X}^{2}}{\sigma_{X}^{2}+\sigma_{\epsilon }^{2}}$ . Using equation (12), the range of sample size is from ${\left(\frac{2\sigma_{\epsilon }^{2}(z_{\alpha }+z_{\beta })}{c-(\mu_{2}-\mu_{1})}\right)}^{2}$ to ${\left(\frac{2(\sigma_{X}^{2}+\sigma_{\epsilon }^{2})(z_{\alpha }+z_{\beta })}{c-(\mu_{2}-\mu_{1})}\right)}^{2}$ . Although the required sample size n is a decreasing function of ρ and ρ is a decreasing function of the time interval between the measurements, we cannot jump into the conclusion that studies with smaller time interval require smaller sample sizes. This is because a smaller time interval usually also results in a smaller difference between $\mu_{1}$ and $\mu_{2}$ .

### QIB as predictive biomarker

In addition to serving as clinical trial end points, QIBs can also be used as predictive biomarkers for early prediction of treatment effect, or as intermediate endpoints in multiarm, multistage trials.^50^ Under this scenario, it is usually assumed that the true value of QIB is associated with the primary trial end point but we only measure QIB with error (see model (1)). Many methods have been proposed to adjust the measurement error on covariates in regression models.^51–53^ However, using such adjustment requires knowledge of the distribution of measurement errors that can only be obtained from additional studies such as repeatability or reproducibility studies. This requirement, on the one hand, emphasizes the importance of repeatability or reproducibility studies, but on the other hand, may not always be applicable and standard approach that ignores the measurement error is conducted.^54^ In this study, we use simulation to illustrate the impact of measurement error when standard approach is used.

Because the sample sizes in published studies where QIBs were used as predictive biomarkers are usually small, it is difficult to analytically evaluate the impact of measurement error on QIBs as predictive biomarkers. Based on the study design and assumptions, Monte Carlo simulations can be used to numerically approximate the impact of measurement error. For illustration purposes, we designed our simulations based on the study by Tudorica et al,^54^ where DCE-MRI QIBs were used for early prediction of breast cancer response [pathologic complete response (pCR) *vs* non-pCR] to neoadjuvant chemotherapy (NACT). We denoted *Z_i_* as the indicator of pCR and *X_i_* as the true value of a DEC-MRI QIB for subject *i*. The true DCE-MRI QIB *X_i_* was generated from normal distribution. Tudorica et al^54^ provided a list of DEC-MRI QIBs with their means and SDs for pCR and non-pCR patients. Here, we considered the percent change in QIB *K* ^trans^ (transfer rate constant) after the first cycle of NACT relative to baseline (pCR: mean = −64%, SD = 9%; non-pCR: mean = −14%, SD = 41%), which showed the best predictive performance for pCR *vs* non-pCR in that study.^53^ Consistent with the sample size of the study,^53^ we included a total of 28 subjects in this simulation study. Here, without loss of generality, we assumed that the first five subjects are pCR patients and the other subjects are non-pCR patients. As noted above, we can only observe the DEC-MRI QIB with error (model (2)). The best approach was to fit a univariate logistic regression model using the observed DEC-MRI QIB *Y_i_* as covariate, *i.e.*

(13) $$log\left(\frac{P(Z_{i}=1)}{1-P(Z_{i}=1)}\right)=\theta_{0}+\theta_{1}Y_{i}$$

**Figure 4.**
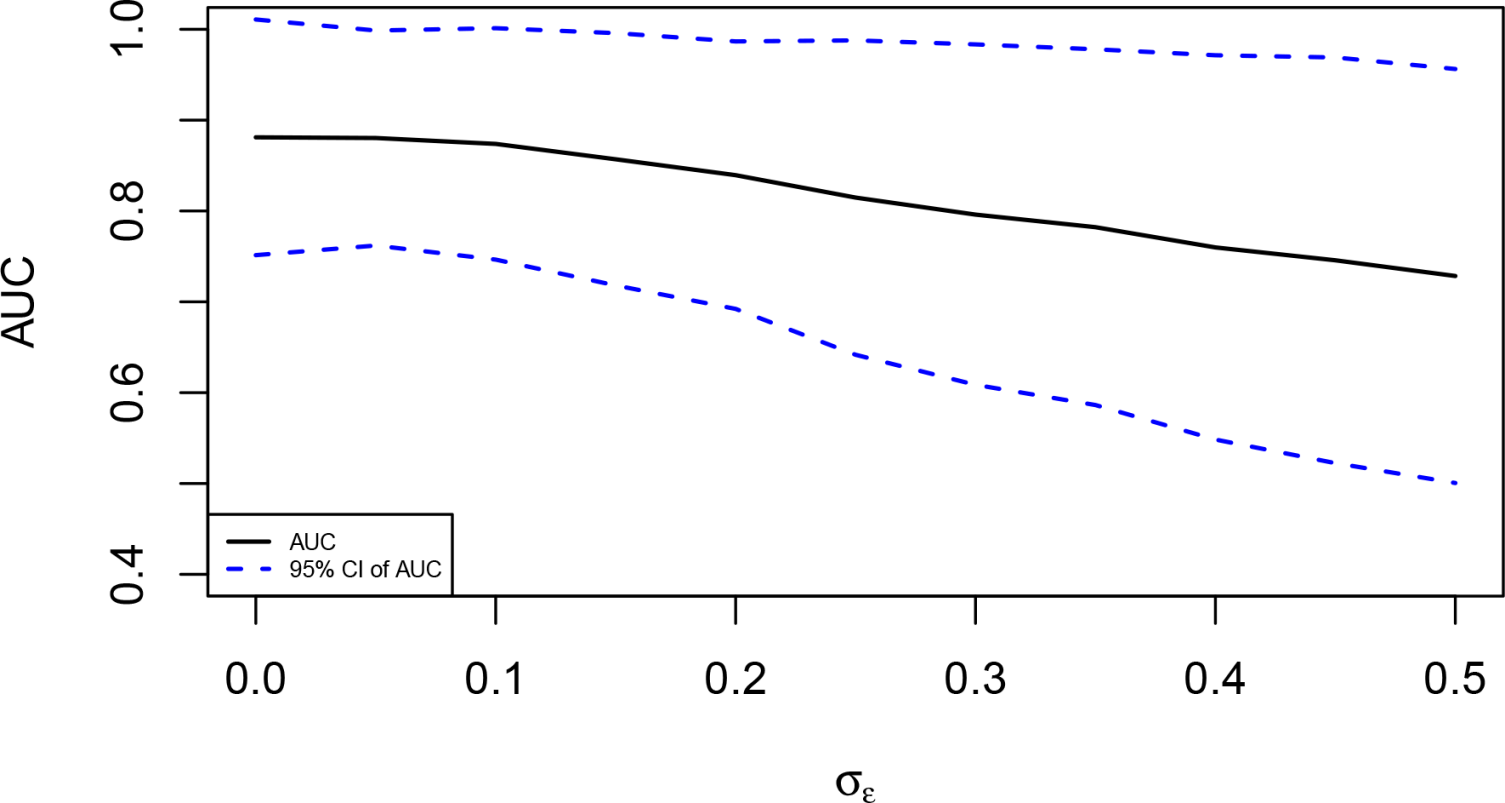
Average AUC (solid black curve) and the corresponding 95% confidence intervals (dashed blue curves) against different values of $\sigma_{\epsilon }$ . Results were obtained based on 1000 simulated data sets. AUC, area under the receiver operating characteristic curve.

Area under the receiver operating characteristic curve (AUC) was used to evaluate the QIB predictive performance. Sample size calculation of AUC can be conducted using the formula provided by Obuchowski et al.^55^ Because the effect of measurement error on AUC is still not clear, we perform a simulation study evaluate this effect. The true *K* ^trans^ percent change values were repeatedly generated 1000 times, and the average AUC across these 1000 simulated data sets and the corresponding 95% CIs were calculated. Figure 4 illustrates the average AUCs against different values of $\sigma_{\epsilon }$ ( $\sigma_{\epsilon }=5\%,10\%,\cdots ,30\%$ ), the measurement error standard deviation in *K* ^trans^ percent change. Our simulation results show that the predictive performance as measured by the average AUC decreases, and the length of 95% CIs increases with increased measurement error ( $\sigma_{\epsilon }$ ).

### Discussion

In this review article, we provided a general introduction on the study design, statistical model, and statistical metrics that can be used to assess repeatability and reproducibility of QIB measurements. We also illustrated the impact of repeatability- and reproducibility-related QIB measurement errors on QIB applications, *e.g.* on sample size calculation when a QIB is used as a clinical trial end point.

The statistical models presented here assume that the measurement errors are normally distributed and independent from the true QIB values. If the measurement errors increase in proportion to the true QIB values, *i.e.* multiplicative errors (see model (7)), log-transformed QIB values can be used as the relationship between error and true value becomes additive after the transformation. In practice, when QIB measurements have values equal or close to zero, we can add a small amount on all QIB values before taking the log-transformation. For more complex error structures such as non-Gaussian or heterogeneous measurement errors, the statistical methods introduced in this article can provide a reasonable approximation of the repeatability and reproducibility metrics of interest, *e.g.* wSD and ICC, but statistical inferences on these estimates can be biased and may lead to false conclusions.

Test–retest studies are commonly used to study repeatability and reproducibility of a QIB, where each object, *e.g.* a phantom or a human subject, is being repeatedly measured. This approach may sometimes be impractical for human subject studies for reasons such as costs, time, and the invasiveness of the imaging scan. As an alternative strategy, Obuschowski et al^32^ proposed a method to estimate the measurement error under repeatability condition when a test–retest study is not feasible. The method requires a reference (golden standard) value is available for each subject. By assuming the reference value to be the true QIB value ( $X_{i}$ ), it can serve as the second measured value for wSD or wCV estimation:

$$\hat{mSD}=\sqrt{\frac{1}{n}\sum_{i=1}^{n}‍{\left(Y_{i1}-X_{i}\right)}^{2}}$$

$$\hat{mCV}=\sqrt{\frac{1}{n}\sum_{i=1}^{n}‍\frac{{\left(Y_{i1}-X_{i}\right)}^{2}}{X_{i}^{2}}}$$

Repeatability and reproducibility can be part of the same study. It is possible to study repeatability in a restricted subset of a reproducibility study to ensure repeatability is acceptable, *e.g*. in an initial subset of subjects going into the study to ensure it is worth pursuing.

There is increasing need to accelerate clinical translation of QIBs. However, significant challenges remain. Using solid tumor therapy response as an example, the 1D imaging tumor size measurement based on the RECIST (Response Evaluation Criteria In Solid Tumors) 1.1 guidelines^56^ is the only widely used QIB in today’s standard of care and clinical trials. Many QIBs that interrogate tumor biology and physiology and thus are well suited for evaluation of response to increasingly used and effective molecular targeted therapies find it difficult to be translated into clinical trials and practice. This is mainly due to the variabilities in quantifying these QIB parameter values caused by differences in vendor imaging platforms, imaging data acquisition methods, and imaging data analysis algorithms and software tools. Because of the lack of sufficient repeatability and reproducibility studies to understand the variabilities of these functional QIBs, unlike RECIST tumor size measurement, there is currently no consensus on the magnitudes of changes in these QIBs for defining clinical response endpoints such as complete response, stable disease, etc. In order to establish a path to clinical translation for functional QIBs, there is a clear need for not only standardization of data acquisition and analysis to minimize variability,^1^ but also more efforts in assessment of QIB repeatability and reproducibility.^1^ It is our hope that the statistical tools presented in this article may contribute to this endeavor.
